# 

**Published:** 1847-03

**Authors:** 


*To Readers and Correspondents.*—Communications have been received from Drs. Reid, Mitchell and Treat, which will appear in our next.

A correspondent informs us that our subscribers at Boston Mass. do notreceive the Journal regularly. We cannot understand how this occurs, as the numbers are all regularly mailed. We will, however, cheerfully have back nos. sent on being notified for what months they have not been received.

---

Dr. I. Lawrence Smith, one of the former Editors of the Southern Journal of Medicine and Pharmacy, has received the appointment of Geologist and Inspector of mines to the Sultan of the Ottoman Empire.

---

*Buffalo Medical College.*—It is not with a little gratification that we announce the auspicious commencement of the first course of Lectures at this institution. Sixty-six have registered as students, a number, which, when it it is considered that the cash system has been rigidly enforced, affords a satisfactory earnest of the future success of the enterprise.

